# Time of In Vitro Anther Culture May Moderate Action of Copper and Silver Ions that Affect the Relationship between DNA Methylation Change and the Yield of Barley Green Regenerants

**DOI:** 10.3390/plants9091064

**Published:** 2020-08-19

**Authors:** Piotr T. Bednarek, Renata Orłowska

**Affiliations:** Department of Plant Physiology and Biochemistry, Plant Breeding and Acclimatization Institute—National Research Institute, Radzików, 05870 Błonie, Poland; r.orlowska@ihar.edu.pl

**Keywords:** AgNO_3_, androgenesis, CuSO_4_, green plants, methylation changes, time

## Abstract

Plant anther culture allows for the regeneration of uniform and homozygous double haploids. However, off-type regenerants may appear as a result of so-called tissue culture-induced variation (TCIV). In addition, the presence of Cu^2+^ and Ag^+^ ions in the culture medium might influence the number of green plants. The regenerants were obtained via anther cultures of barley under varying Cu^2+^ and Ag^+^ ion concentrations in the induction medium during distinct time conditions. DArTseqMet markers were evaluated based on regenerants and donor plants and delivering data on DNA demethylation (DM) and de novo methylation (DNM) and changes in methylation (Delta). The number of green regenerated plants per 100 anthers (GPs) was evaluated. The Cu^2+^ and Ag^+^ ion concentrations moderated relationships between Delta and the number of green plants conditional on time of tissue cultures. Depending on the ions, moderated moderation is valid within the different time of anther culture. When the highest concentration of copper is analyzed, plant regeneration is possible under short ‘Time’ (21 days) of anther culture wherein Delta is negative or under elongated Time when Delta is positive. Under 21 days of culture, the highest concentration of silver ions and when Delta is negative, some regenerants could be evaluated. However, under high Ag^+^ concentration when Time of culture is long and Delta positive, the highest number of green plants could be obtained.

## 1. Introduction

Plant regeneration via anther cultures requires precise tuning of the in vitro tissue culture conditions, including the concentration of ingredients (i.e., the balanced concentration of ions) that may influence cellular processes promoting plant regeneration. Such ingredients may encourage the induction of cell reprogramming [1,2,3,4] and potentially change the balance of biochemical pathways. For example, the addition of Cu^2+^ ions may affect mitochondrial Complex IV [5] belonging to the electron transport chain and is involved in the copper-delivery pathway and creates functional ethylene receptors [6]. It may also form complexes with ethylene. Its improper functioning may result in ATP deficiency [7,8], or burst of reactive oxygen species (ROS) [9]. Copper ions are responsible for generation of hydroxyl radicals, which may attack the DNA bases in a site-specific manner [10,11]. In addition, copper ions may interact directly with the DNA or affect the electrophoretic mobility of the molecule [12]. Copper ligands are known to disturb DNA helix resulting in DNA conformational changes and DNA breakages [13] as indicated by docking studies [14]. They are also considered as putative drugs against cancer [15]. On the other hand, silver ions regulate the polyamine pool in a plant [16], ethylene- and calcium-mediated pathways and plays a role in the physiological process [17]. Some of the pathways depending on copper or silver ions present in the regeneration medium may affect nuclear DNA [18] at the methylation level [19]. As DNA methylation is required for microspore reprogramming towards sporophytic path thus, changes in gene expression pattern may influence green plant regeneration.

To study the DNA methylation changes and their relation with the number of green plants, several molecular marker systems could be exploited, including methylation sensitive Amplified Fragment Length Polymorphism (metAFLP) [20], Methylation Sensitive Amplification Polymorphism (MSAP) [21] and Diversity Arrays Technology (DArT) [22]. The first two are based on the AFLP approach [23] but use distinct isoschizomers recognizing different restriction sites and different methylation patterns [24,25]. A newly developed DArT system (DArTseqMet—Diversity Arrays Technology Methylation Analysis) that takes advantage of methylation changes due to HpaII and MspI isoschizomers in combination with Next Generation Sequencing (NGS) [26,27] might also be a method of choice. The results of the DArTseqMet could be used for quantification of the DNA methylation changes if a semi-quantitative MSAP technique is involved [28].

Finally, to study relationships of different factors affecting the given phenomenon, moderation and mediation analysis could be employed [29]. The approach is mostly used in psychology [30], medicine [31], economy and business sciences [32]. It allows the identification of moderators or mediators; however, it was hardly used in studies on in vitro tissue cultures [33]. It seems that it may have a wide range of applications allowing a better understanding of relations among many factors of biological systems.

Our previous studies have demonstrated that manipulating Cu^2+^, Ag^+^ ion concentrations one may optimize in vitro anther culture of barley towards the increased number of green plants [34]. We have also shown that under varying conditions of Cu^2+^, Ag^+^ and time of in vitro tissue cultures differences in DNA methylation change may appear [35]. According to that result, the role of time seems to be negligible, whereas the others [36] indicated that the longevity of in vitro tissue cultures might influence DNA methylation patterns [37] or even sequence changes [38]. We suspect that time of in vitro tissue cultures may moderate the action of the ions being present in the in vitro tissue culture medium affecting relationships between DNA methylation pattern change and the number of green plants regenerated via in vitro anther cultures. Moreover, we cannot exclude that Cu^2+^ and Ag^+^ ions present in the induction medium act simultaneously promoting green plant regeneration in anther cultures.

## 2. Results

In vitro anther tissue cultures performed under nine distinct conditions (trials M1–M9) varying in the Cu^2+^, Ag^+^ ion concentrations and time (Time) allowed the regeneration of 35 plants. No callus was observed during the tissue culture until day 21, after which callus was evidenced. The phenotype of the obtained regenerants, taking into account such features as leaf height, width or the method of tillering, was identical to that of the donor plants from which explants were taken to establish anther cultures. DNA isolation from fresh leaves of donor and regenerated DH plants using commercial kits resulted in integral samples without impurities. The new generation sequencing approach exploiting HpaII and MspI endonucleases that differ in sensitivity towards site DNA methylation [39] was used to evaluate DArTseqMet DNA markers. The markers were classified to those related to de novo methylation (DNM) and demethylation (DM), following the procedure described earlier [28]. The DNA methylation characteristics including DM, DNM and changes in methylation (Delta = DM-DNM), and the number of green plants regenerated per 100 anthers (GPs) within each trial was evaluated, as indicated in Table 1.

We were interested in how Time (Z, see section Material and Methods, Figure 3) of anther culture influences the extent to which Cu^2+^ and Ag^+^ (W) ions moderate relationships between a Delta (defined as DM-DNM) predictor being and independent variable (X) and the number of green plants regenerated per 100 anthers (GPs) (the outcome variable—Y). By employing a three-way interaction model, we estimated the moderating influence of Time, which operates as a secondary moderator [29]. In the copper ions, all the predictors of the moderated moderation appeared significant (Table 2). Nearly the same is valid for silver ions except for Time (Table 2). The overall three-way interaction model for copper and silver accounted for 98 and 92% of the total variance explaining the GPs by the Delta (*F(7.27)* = 249.55, *p* < 0.001) and Delta (*F(7.27)* = 46.99, *p* < 0.0001), respectively. Furthermore, the three-way interaction of Delta by Time and by Cu^2+^ as well as Delta by Time and by Ag^+^ accounted for 29.7 (*F(1,27)* = 527.15, *p* < 0.001) and 61.7% (*F(7.27)* = 46.99, *p* < 0.0001) of the variance. The moderated moderation model showed significant and positive three-way interaction between Delta, Time and Cu^2+^ and Ag^+^, (*β* = 0.03, *SE* = 0.001, *p* < 0.001) (Table 2) and (*β* = 0.021, *SE* = 0.018, *p* < 0.001) (Table 3). Johnson–Neuman’s statistics indicated that conditional effects of the focal predictor (Delta) at values of the moderator (Cu^2+^, Ag^+^) were significant within 21–23.1 and 25.2–35 days for copper and 21–28 and 29.4–35 days of an anther tissue culture of barley for silver.

**Figure 1:**
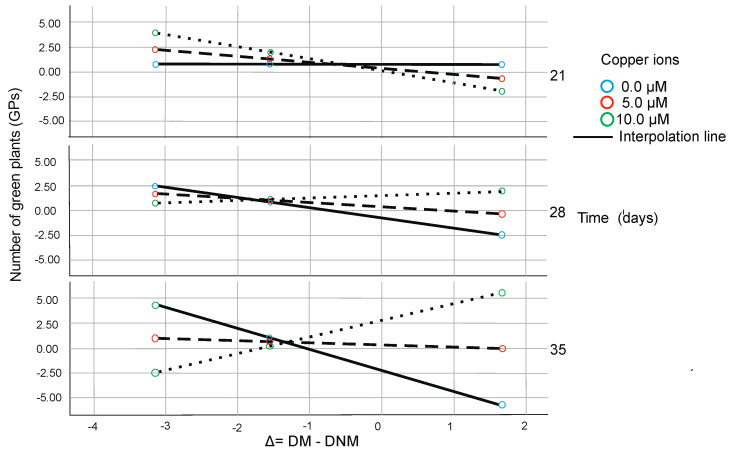
The conditional effect of Delta on the number of green plants (GPs) derived per 100 spikes as a function of copper ion concentration and Time of in vitro anther culture from moderated moderation model. Category axis (axis of abscissa) is indicated as Delta (∆). It illustrates values of Delta expressed as DM-DNM in percentage. Axis of ordinates reflects the number of green plants (GPs) regenerated per 100 anthers expressed in a number of regenerants. Blue, red and green circles indicate the different concentrations of copper ions.

**Figure 2:**
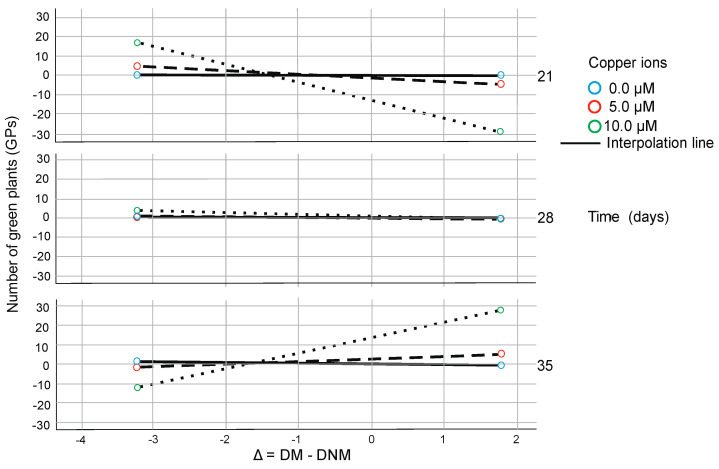
The conditional effect of Delta on the number of green plants (GPs) derived per 100 spikes as a function of copper ion concentration and Time of in vitro anther culture from moderated moderation model. The category axis (axis of abscissa) is indicated as Delta (∆). It illustrates the values of Delta expressed as DM-DNM in percentage. Axis of ordinates reflects the number of green plants (GPs) regenerated per 100 anthers expressed in a number of regenerants. Blue, red and green circles indicate the different concentrations of silver ions.

The effect of Delta on the number of green plants depends on copper and silver ion concentration and Time of anther culture. Under 21 days of culture and zero copper ions concentration, it is insignificant through the whole delta spectrum. However, it is becoming negative when Time increases. When the highest concentration of copper is analyzed, plant regeneration is possible under short Time (21 days) of anther culture when Delta is negative or under elongated Time when Delta is positive. With Time (under the highest copper ion concentration), positive Delta results in the highest number of green regenerants (Figure 1). Under 21 days of culture, the highest concentration of silver ions, when Delta is negative, some regenerants could be evaluated. However, under high Ag^+^ concentration when Time of culture is long and Delta positive, then the highest number of green plants could be obtained. When the lowest concentration of silver is analyzed, plant regeneration is impossible through the whole Time of anther culture independently of Delta values. With Time (under the highest copper ion concentration), the negative effect of Delta changes from negative to positive and results in the highest number of green regenerants (Figure 2).

The effect of Delta on the number of green plants depends on copper ion concentration and Time of anther culture. Under 21 days of culture and zero concentration of ions, it is insignificant through the whole delta spectrum. However, it is becoming negative when Time increases. When the highest concentration of copper is analyzed, plant regeneration is possible under short (21 days) Time of anther culture when Delta is negative or under elongated Time when Delta is positive. With Time (under the highest copper ion concentration), the negative effect of Delta changes from negative to positive and results in the highest number of green regenerants (Figure 1).

The effect of Delta on the number of green plants also depends on silver ion concentration and Time of anther culture. Under 21 days of culture and the highest concentration of ions, it is negative. However, it is becoming positive when Time increases and results in the highest number of green plants. When the lowest concentration of silver is analyzed, plant regeneration is not possible through the whole Time of anther culture independently of Delta values. With Time (under the highest copper ion concentration), the negative effect of Delta changes from negative to positive and results in the highest number of green regenerants (Figure 2).

Box’s test of equality of covariance matrix was used to verify whether covariance matrices among dependent variables are the same for all groups (MANOVA assumption). Based on the test significance (Box’s *M = 176.08, F(6,18201.872) = 26.646, p < 0.005*), the MANOVA assumption was violated. The least sensitive towards violation of covariance matrices equality is Pillai’s Trace test. Based on multivariate test, a statistically significant difference in DM and DNM based on Time of in vitro tissue culture (*F(4, 64) = 4.57, p = 0.003*; Pillai’s trace *= 0.44*) was shown.

The ANOVA assumption of homogeneity of variances verified by Levene’s statistics showed that in the case of DM and DNM, homogeneity was violated. As homogeneity assumption was violated, the robust test of equality of mean (Welch statistics) was implemented. Analysis of variance (ANOVA) where demethylation (DM) or de novo methylation (DNM) were dependent and Time an independent one, showed statistically significant differences between group means as indicated by Welch statistics (*F(2, 18.97) = 4.823, p = 0.02* and *F(2, 18.26)* = 6.567, *p = 0.007* for DM and DNM, respectively) were significant. The eta-squared coefficients for the first and the second DNA methylation characteristics equaled to 0.253 and 0.253, respectively. Based on post hoc test (Duncan), DM and DNM characteristics grouped regenerants derived after the 28th day in a single group, whereas the other regenerants into the second group (Table 3). Additionally, to confirm differences among groups of data in the case of DM and DNM, we have applied the Kruskal–Wallis h test that does not require normal distribution. According to the test the differences in the case of DM was not (χ^2^(2) = 4.68, *p* < 0.09) whereas in DNM it was (χ^2^(2) = 11.64, *p* < 0.01) significant.

## 3. Discussion

In vitro anther tissue cultures is an essential approach for the evaluation of uniform plant materials useful for breeding programs [40,41,42,43]. The approach is being world-wide used, but a growing number of data clearly shows that plant regeneration via anther culture is affected by an in vitro tissue culture-induced variation (TCIV) [44,45,46,47] that could be transmitted to a progeny [48]. Among others, the TCIV is due to changes in the DNA methylation patterns that are under either genetic or epigenetic control [49]. As plant regeneration requires cell reprogramming [18] involving DNA methylation pattern change, it is of value to understand whether ingredients present in the in vitro medium participating in many cellular biochemical pathways [17,50,51] and other factors such as time of in vitro cultures may affect methylation changes and the number of the regenerated green plants that may be limited by the presence of albinos [52]. Among many compounds used in the in vitro anther culture, Cu^2+^ and Ag^+^ are being considered as promising [53,54]. There are shreds of evidence that the ions may increase the number of green regenerants in cereals [55,56]. However, the action of the ions may depend on their concentration in the medium as the cell wall components, i.e., cellulose may absorb them limiting their availability for the proper functioning of the respiration pathway [57]. However, the role of the ions needs further studies.

The moderation analysis demonstrated that copper and silver ions present in tissue culture of barley affect the relationship between demethylation vs. de novo methylation change and the number of GPs. First, regenerants derived in tissue cultures were evaluated after 21 days of tissue culture under the highest value of the Cu^2+^ and Ag^+^ ions. At this stage, the Delta is negative, meaning that DM is lower than DNM, indicating that DNA methylation is reestablished after the reprogramming stage. It should be stressed that after 21 days of in vitro tissue culture, the first visual evidence of spherical structures that resulted in regenerants was seen. To some extent, the appearance of such structures corresponded to the action of copper ions that started to be insignificant moderators of plant regeneration through 23.1–25.2 days of tissue culture. As direct embryogenesis proceeds indirect one (the most probable path), it is suggested that the GPs regenerated by this time originated via direct embryogenesis from microspores. The notion corresponds to the experimental design of barley anther cultures run on the KBP medium, which favors direct embryogenesis. From this point of view, the observation that increasing the time of tissue culture resulted in the increase of calli (differed in appearance from spherical structures) becomes apparent. Our results agree with the data presented by others [49], showing that the very first regenerants originated after 21 days of tissue cultures, and new regenerants arrived more or less at a constant number for 35 days. Thus, time conditions under which moderated moderation due to copper ions is not significant may reflect the moment when callus rather than regeneration medium becomes a preferential feeding source for new regenerants. However, the respective sources need to be available.

It was demonstrated that in darkness, β-glucans could be exploited as a source of glucose in cereals [58]. It was suggested [59] that glucose from the wall in a form of (1,3;1,4)-β-glucans could be mobilized quicker than glucose from starch. Moreover, the data on the concentration of (1,3;1,4)-β-glucans near vascular bundles in barley leaves are consistent with the ability to transport glucose released from leaves to other parts of the plant [59]. Possibly, to some extent, the same phenomenon takes place in tissue culture when callus is being developed. The availability of glucose from β-glucans and rapid transport to the regions of plant regeneration may be an important aspect of indirect embryogenesis delivering glucose for new regenerants. The phenomenon when there is an increase in the number of new regenerants after a prolonged time of in vitro culture may be explained by the various origin of developed embryos (direct and indirect somatic embryogenesis) [60].

Interestingly, silver ions’ action does not necessarily overlap with those of copper once with a shift of insignificant moderation of the relationship between Delta and GPs by silver ions to 28 to 29.4 days of culture. Such a shift is not evident; however, it may reflect the time required for preferential substitution of Cu^2+^ by silver [61] in the respiratory chain [62]. MANOVA supports such a notion. We have demonstrated that depending on time, the regenerants differed in DM and DNM. ANOVA followed by the Duncan test based on DM and DNM grouped regenerants derived before and after about the 28th day of tissue culture. Similar results were evaluated using the Kruskal–Wallis h test for DNM. Moreover, the 28th day of tissue culture is when moderated moderation of silver ions is insignificant. We tend to interpret the result in terms of disturbances of the methionine cycle responsible for DNA methylation [63] by silver ions that become detectable after a longer time of tissue cultures.

The limitation of the current study is a relatively small sample size. Running tissue culture experiment under restrictive conditions (only regenerants derived from a single DH plant were used to fit the genetic analysis) results in a limited number of regenerants. However, all coefficients were tested via bootstrapping, to test whether the presented moderation model is significant. A test is an adequate option for such circumstances even if the sample size varies from 20 to 89 [64,65]. As all the coefficient passed the test, we assumed that the presented model is significant. Moreover, the model agrees with the data indicating that Cu^2+^ and Ag^+^ may influence the number of GPs [34] and the fact that both ions are of comparable sizes [66] and Cu^2+^ may be substituted by Ag^+^ [61] in mitochondrial complex III [62]. Thus, we tend to think that despite the sample size, the presented moderated moderation model reflects a real phenomenon. Nevertheless, further studies are needed to confirm whether it could be extended on other species.

The presented data demonstrate that implementation of statistics in tissue culture studies may result in constructing theoretical models illustrating biological phenomena that might be of value for better understanding in vitro anther culture processes affecting plant regeneration. The approach is especially valuable when many variables are to be combined in a single analysis as was demonstrated by us earlier using mediation analysis [33]. Unfortunately, such experiments are rather rare due to problems related to the number of available samples, proper models for review, or the necessity of implementation of expensive molecular markers and their quantification.

## 4. Materials and Methods

### 4.1. Plant Material

Plant materials (spring barley cultivar NAD2 was provided by Poznan Plant Breeders LTD-Nagradowice, Poland) were obtained, as described earlier [34]. Briefly, spikes of donor doubled haploid (DH) plants of barley were used to regenerate new DH plants under varying conditions of Cu^2+^ and Ag^+^ ion concentrations and time of tissue cultures. Nine of such trials were performed (M1-M9). Each trail characterizes varying concentrations of CuSO_4_ and AgNO_3_ that were added to the induction medium, as shown in Table 4. Time from plating the anthers on induction media to collecting the callus, determined as time of tissue culture (Time), was different for each trial (Table 4). Trail M1 served as a control for the tissue culture conditions examined in the tested trials (M2-M9). Finally, 11,722 anthers were plated onto induction media throughout the experiment, without being divided into individual trials.

DNA isolation was performed from fresh leaves of donor and regenerated plants using the DNeasy MiniPrep kit (Qiagen, Hilden, Germany). DArTseqMet was conducted in DArT PL company (DArTseqMet, developed by Diversity Arrays Technology, https://www.diversityarrays.com/technology-and-resources/dartseq) on 35 regenerants. DArTSeqMet markers were converted into semi-quantitative methylation characteristics following the MSAP approach [28]. Briefly, a comparison of molecular data generated due to genome complexity reduction via HpaII and MspI endonucleases following genetic interpretation of such data allowed their assignment to specific events, their quantification and development of MSAP characteristics reflecting DNA methylation changes.

### 4.2. Moderated Moderation Analysis

**Figure 3:**
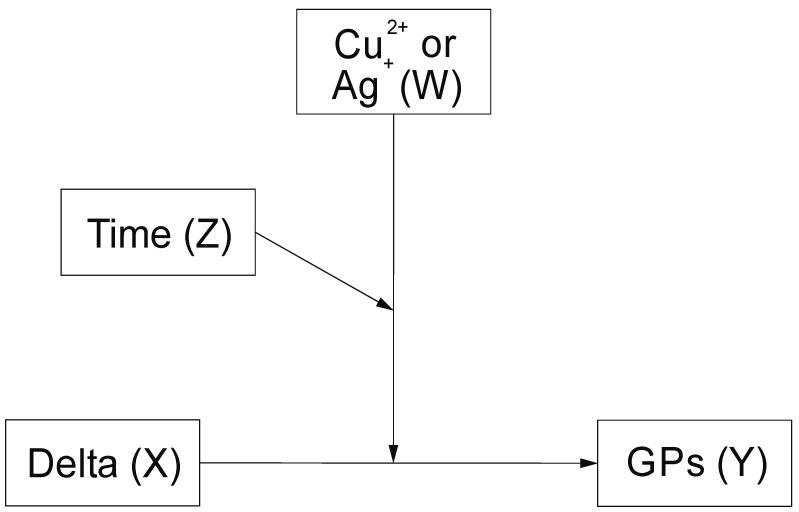
Schematic illustration of conditional moderation model. X is a Delta (DM-DNM), Y is the outcome variable reflecting the number of green plants regenerated per 100 anthers (GPs), W states for Cu^2+^ or Ag^+^ ions conditional on Time of in vitro tissue culture (Z).

Moderation analysis describes relationships between some variables (X and Y) that are influence by the other one (W) that changes the strength of their relationships. In some cases, the strength of the moderator (W) may be affected by a second factor (Z). In such a case moderated moderation model is assumed. The analysis was conducted in SPSS software V. 26 (https://www.ibm.com/support/pages/node/874712) using A. F. Hayes Process v. 3.4 macro [29]. Moderated moderation model nr 3 (Figure 3) was tested.

### 4.3. Analysis of Variance

The presence of significant differences among combinations of levels of factors (Time of anther culture) on DM and DNM response variables was verified by multivariate analysis of variance (MANOVA) using Pillai’s test. MANOVA assumption of covariance matrices similarity was tested using Box’s test. The analysis was conducted in SPSS software v.26.

Differences among regenerants derived after 21, 28, and 35 days were evaluated using Analysis of Variance (ANOVA). Levene statistics were used to test for homogeneity of variance. Shapiro–Wilk test was applied to verify normal distribution. A robust test of equality of means (Welch statistics) followed by the Post hoc test (Duncan) was calculated. Non-parametric Kruskal–Wallis h test (K-independent samples) was applied in parallel to ANOVA. The calculations were conducted in SPSS v 26. The effect size measure (η2—eta-squared) was calculated in excel as the ratio of the effect variance (SS_effect_) to the total variance (SS_total_).

## 5. Conclusions

The main conclusion from the current study is that copper and silver ions play in concert moderating relationships between Delta and GPs. The cooperative effect of silver and copper ions is most probably due to the fact that silver ions may substitution copper ones as they are both from Group 11 of the periodic table. Moreover, the action of the ions is affected by time of in vitro anther culture of barley. It is worth mentioning that in order to regenerate green plants, high values of variable reflecting combined copper and silver ions is required.

## Figures and Tables

**Table 1:** The arrangement of de novo DNA methylation (DNM) and DNA demethylation (DM) quantitative characteristics evaluated based on DArTseqMet markers exploiting a semi-quantitative Methylation Sensitive Amplification Polymorphism (MSAP) approach using DNA samples of anther culture regenerants derived under the varying concentration of copper and silver ions in the medium and under distinct time of anther cultures (trials M1–M9). The number of green plants regenerated per 100 anthers (GPs) under the nine trials is also indicated, whereas Delta is the difference between demethylation and de novo methylation (DM-DNM). Mean (*M*) and standard deviation (*SD*) are also indicated.

Regenerant	Trial	DNM (%)	DM (%)	Delta (%)	GPs
1	M1	28.153	32.179	4.026	0.64
2	M1	28.188	32.289	4.101	0.64
3	M1	28.155	32.247	4.092	0.64
4	M1	28.135	32.172	4.037	0.64
5	M1	28.102	32.209	4.107	0.64
6	M2	31.094	29.429	−1.665	0.67
7	M2	31.095	29.583	−1.512	0.67
8	M2	31.005	29.457	−1.548	0.67
9	M3	30.915	29.288	−1.627	1.09
10	M3	30.855	29.301	−1.554	1.09
11	M3	30.876	29.332	−1.544	1.09
12	M3	30.915	29.276	−1.639	1.09
13	M3	30.920	29.309	−1.611	1.09
14	M4	30.421	30.532	0.111	0.45
15	M4	30.457	30.591	0.134	0.45
16	M5	6.897	7.834	0.937	0.1
17	M5	10.804	11.344	0.540	0.1
18	M5	6.825	7.609	0.784	0.1
19	M5	11.043	11.170	0.127	0.1
20	M6	31.524	28.674	−2.850	2.12
21	M6	31.295	28.650	−2.645	2.12
22	M6	31.505	28.567	−2.938	2.12
23	M6	31.517	28.674	−2.843	2.12
24	M6	31.355	28.815	−2.540	2.12
25	M7	29.909	29.972	0.063	2.91
26	M7	29.999	30.106	0.107	2.91
27	M7	29.979	29.991	0.012	2.91
28	M8	31.260	29.659	−1.601	1.77
29	M8	31.275	29.784	−1.491	1.77
30	M8	31.249	29.786	−1.463	1.77
31	M9	32.197	28.127	−4.07	0.54
32	M9	32.265	28.277	−3.988	0.54
33	M9	32.234	28.225	−4.009	0.54
34	M9	32.195	28.308	−3.887	0.54
35	M9	32.192	28.286	−3.906	0.54
*M*		28.196	27.402	0.794	1.123
*SD*		7.17	6.67	2.47	0.85

**Table 2:** The moderated moderation model showing three-way interaction among Delta, Time of in vitro tissue cultures and Cu^2+^ (Ag^+^) ion concentration on the number of green plants regenerated via anther cultures. *β* reflect standardized effects; LLCI and ULCI indicate confidence intervals (LLCI -lower, UPLCI upper) whereas SE is a standard error of *β*.

Predictors	Statistics			
	*β*	*SE*	95% LLCI	95% ULCI
Delta = DM-DNM	3.06 ***(0.079 ***)	0.12(0.19)	2.812(0.323)	3.310(1.096)
Cu^2+^ (Ag^+^)	−0.93 ***(−0.87 ***)	0.05(0.06)	−1.031(−0.987)	−0.837(−0.753)
Delta × Cu^2+^ (Ag^+^)	−0.86 ***(−0.606 ***)	0.04(0.04)	−0.950(−0.689)	−0.771(−0523)
Time	−0.21 ***(0.017)	0.01(0.01)	−0.229(−0.007)	−0.192(0.040)
Delta × Time	−0.15 ***(−0.028 ***)	0.01(0.00)	−0.157(−0.044)	−0.136(−0.12)
Cu^2+^ (Ag^+^) × Time	0.04 ***(0.031 ***)	0.01(0.00)	0.038(0.027)	0.044(0.035)
Delta × Cu^2+^ (Ag^+^)^+^ × Time	0.03 ***(0.021 ***)	0.00(0.00)	0.032(0.018)	0.038(0.024)
Model Summary for Cu^2+^ (Ag^+^)	*R^2^ = 0.985, F (7,27) = 249.55, MSE = 0.014, p < 0.001* *(R^2^ = 0.924, F (7,27) = 46.99, MSE = 0.014, p < 0.001)*			
Test of highest order unconditional interaction (X × W × Z: Delta × Cu^2+^ (Ag^+^) × Time)	*F(1,27) = 527.15, R^2^_chng_ = 0.297, p < 0.001* *(F(1,27) = 219.76, R^2^_chng_ = 0.617, p < 0.0001)* *** p < 0.001			

**Table 3:** The arrangement of Duncan test results concerning differences in DM and DNM due to the days of tissue culture.

Days	DM ^1^	DNM ^1^
21	30.29 ^a^	30.13 ^a^
28	29.08 ^ab^	31.52 ^a^
35	22.88 ^b^	23.33 ^b^

^1, a^ and ^b^ reflect grouping by the Duncan test.

**Table 4:** Concentrations of ions and number of days adopted in subsequent trials (M1–M9).

Regenerant	Trial	Cu^2+^ (µM)	Ag^+^ (µM)	Time (Days)
1–5	M1	0.1	0	21
6–8	M2	0.1	10	28
9–13	M3	0.1	60	35
14–15	M4	5	60	28
16–19	M5	5	0	35
20–24	M6	5	10	21
25–27	M7	10	10	35
28–30	M8	10	60	21
31–35	M9	10	0	28

## Abbreviations

Delta	change between DM and DNM, Delta = DM - DNM
DArT	Diversity Arrays Technology
DArTseqMet	Diversity Arrays Technology Methylation Analysis
DM	demethylation
DNM	de novo methylation
GPs	number of green plants regenerated per 100 anthers
IUPAC	International Union of Pure and Applied Chemistry
metAFLP	methylation sensitive Amplified Fragment Length Polymorphism
MSAP	Methylation Sensitive Amplification Polymorphism
NGS	Next Generation Sequencing
ROS	reactive oxygen species
TCIV	tissue culture-induced variation
